# A cucumber green mottle mosaic virus vector for virus-induced gene silencing in cucurbit plants

**DOI:** 10.1186/s13007-020-0560-3

**Published:** 2020-02-03

**Authors:** Mei Liu, Zhiling Liang, Miguel A. Aranda, Ni Hong, Liming Liu, Baoshan Kang, Qinsheng Gu

**Affiliations:** 1 Zhengzhou Fruit Research Institute, Chinese Academy of Agricultural Sciences, Zhengzhou, 450009 People’s Republic of China; 2 Huazhong Agricultural University, Wuhan, 430070 People’s Republic of China; 3 Centro de Edafologia y Biologia Aplicada del Segura (CEBAS)-CSIC, Apdo. Correos 164, Espinardo, 30100 Murcia, Spain

**Keywords:** Cucumber green mottle mosaic virus, Viral vector, Virus-induced gene silencing, Cucurbit plants

## Abstract

**Background:**

Cucurbits produce fruits or vegetables that have great dietary importance and economic significance worldwide. The published genomes of at least 11 cucurbit species are boosting gene mining and novel breeding strategies, however genetic transformation in cucurbits is impractical as a tool for gene function validation due to low transformation efficiency. Virus-induced gene silencing (VIGS) is a potential alternative tool. So far, very few ideal VIGS vectors are available for cucurbits.

**Results:**

Here, we describe a new VIGS vector derived from cucumber green mottle mosaic virus (CGMMV), a monopartite virus that infects cucurbits naturally. We show that the CGMMV vector is competent to induce efficient silencing of the phytoene desaturase (*PDS*) gene in the model plant *Nicotiana benthamiana* and in cucurbits, including watermelon, melon, cucumber and bottle gourd. Infection with the CGMMV vector harboring *PDS* sequences of 69–300 bp in length in the form of sense-oriented or hairpin cDNAs resulted in photobleaching phenotypes in *N. benthamiana* and cucurbits by *PDS* silencing. Additional results reflect that silencing of the *PDS* gene could persist for over two months and the silencing effect of CGMMV-based vectors could be passaged.

**Conclusions:**

These results demonstrate that CGMMV vector could serve as a powerful and easy-to-use tool for characterizing gene function, controlling viral pathogens or even performing resistance breeding in cucurbits. Moreover, this study will possess considerable important reference value for developing different viral vectors.

## Background

The family *Cucurbitaceae* is second only after the *Solanaceae* for its economic importance among horticultural species worldwide, containing about 1000 species in 96 genera [1]. Cucurbits are generally prized for their delicious fruits, which might be low in nutritional value, but can be significant dietary sources of minerals and vitamins, some even with medical values. Watermelon (*Citrullus lanatus*), melon (*Cucumis melo*), cucumber (*Cucumis sativus*) and bottle gourd (*Lagenaria siceraria*) all belong to the family *Cucurbitaceae* with a significant impact on human nutrition [2].

With the increase of consumer's demand for high-quality fruits and vegetables and the improvement of agricultural production, it is urgent to explore genes encoding important agronomic traits in crop species, in order to breed elite, disease-resistant and featured varieties. So far, 11 reference genomes of cucurbit species [3] including watermelon [4], melon [5] and cucumber [6] have been published, which have boosted gene mining and gene function research. However, the genetic transformation of cucurbit plants is time-consuming and labor-intensive, with extremely low efficiencies [7]. As a tool for rapid gene function validation, virus-induced gene silencing (VIGS) is a good alternative to gene transformation because of its simplicity, high efficiency, and high throughput.

Gene silencing comprises transcriptional gene silencing (TGS) and post-transcriptional gene silencing (PTGS). VIGS, a type of PTGS, is a natural defense reaction that exists in a broad range of organisms. It confers resistance to foreign nucleic acid invasion through PTGS at the RNA level. Because it can silence a specific gene, leading to the loss of function of this gene, the potential of VIGS as a tool to analyze gene function has been quickly recognized [8].

In the past decades, a large number of viral vectors had been developed as powerful tools for the functional verification of genes in plants [9–16]. To date, three different RNA viruses have been developed as vectors for VIGS in cucurbit species, including apple latent spherical virus (ALSV) [11], tobacco ringspot virus (TRSV) [17] and tobacco rattle virus (TRV) [18, 19]. However, very few applications of these vectors have been reported, implying that they have not been widely adopted for cucurbit gene function analyses. This might be related to their limited host range among cucurbits, cumbersome inoculation approaches and/or short silencing periods associated with insert instability. As a result, it is urgent to develop a vector with a wider range of cucurbit hosts, ease of inoculation, high silencing efficiency and long-lasting gene silencing in cucurbit plants.

Cucumber green mottle mosaic virus (CGMMV) is an important pathogen infecting cucurbit plants in natural conditions [20]. We have successfully constructed a full-length infectious clone of CGMMV, which can systemically infect plants of various cucurbit species such as watermelon, melon, cucumber and bottle gourd [21], making it a good candidate for VIGS vector development in cucurbits. CGMMV is a member of the genus *Tobamovirus*, and has a positive single-stranded genomic RNA of approximately 6.4 kb [22]. The CGMMV genome possesses four open reading frames (ORFs) encoding two replication-related proteins, one movement protein (MP), and one coat protein (CP). Only the 129 KDa and 186 KDa of replication-related proteins are translated directly from the genomic RNA, whereas the 29-KDa MP and the 17.4-KDa CP are translated from two subgenomic RNAs. There is an overlap between the MP and CP ORFs [22]. Viral vectors based on CGMMV for expressing foreign genes have been constructed. Multiple cloning sites (MCS) were inserted adjacent to the CP ORF, and the CP stop codon was altered to express the hepatitis B surface antigen and a Dengue virus Epitope so that 20 and 44 foreign amino acids, respectively, were expressed [23, 24]. Tobacco mosaic virus (TMV), another member of the genus *Tobamovirus*, has been widely studied as a model in this genus. TMV has successfully been developed as a VIGS vector by including an additional duplicated copy of the CP subgenomic promoter (SGP) in the viral genome [25]. The CGMMV genome is similar to that of TMV, and thus it was thought that methods similar to those used for TMV could be used to create vectors based on CGMMV; unfortunately, results in this regard varied largely [26, 27], therefore the strategy of SGP duplication and information on the subgenomic promoter have not been fully exploited for constructing CGMMV-based viral vectors.

CGMMV has not been reported for its development for VIGS, although it has been exploited as a transient gene expression vector by read through translation or adding an additional subgenomic CP promoter. In this study, we developed a new CGMMV-based VIGS vector, which produces very mild viral symptoms and efficiently triggers gene silencing in the model plant *N. benthamiana* and cucurbit plants such as watermelon, melon, cucumber and bottle gourd.

## Results

### Construction of a set of CGMMV vectors

**Fig. 1:**
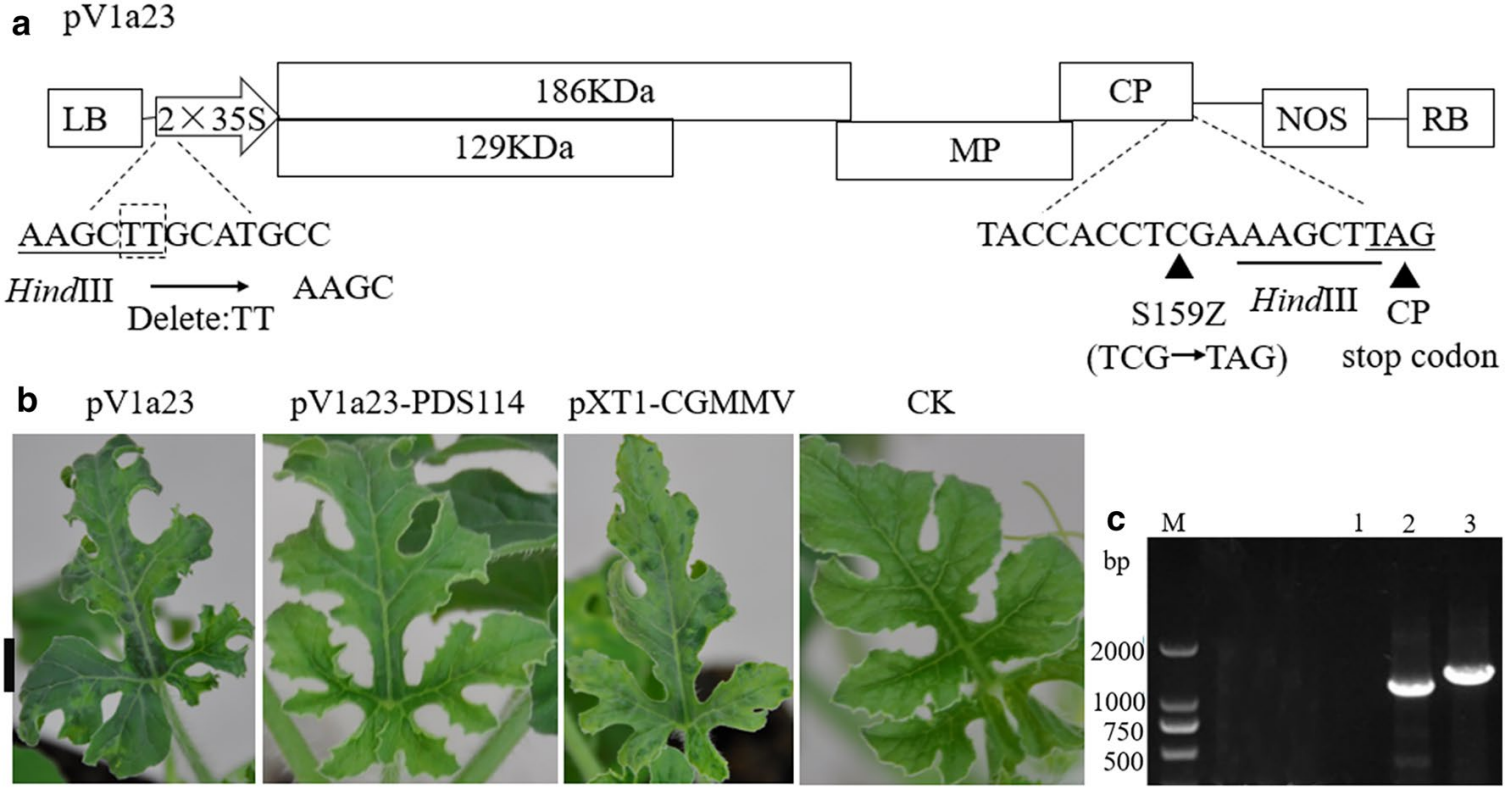
Engineering of CGMMV as a VIGS vector with an insertion site behind the CP. **a** Schematic representation of the pV1a23 vector with a restriction enzyme site (*Hind*III) for insertion of gene fragments. **b** Viral symptoms on upper non-inoculated leaves caused by pV1a23 similar to those of plants inoculated with the pXT1-CGMMV. Photobleaching was absent on plants inoculated with pV1a23-PDS114. Bar = 1 cm. **c** RT-PCR detection of viral RNA from pV1a23 and pV1a23-PDS114 in watermelon. M, Marker2000; CK, negative control; 1, 2 and 3 indicate healthy control and plants inoculated with pV1a23 and pV1a23-PDS114, respectively

The first step in constructing a VIGS vector is to determine the insertion site of a foreign gene fragment. For CGMMV, we can place the insertion site behind the viral MP gene or between the CP gene stop codon and the 3′ non-coding region. For the set of vectors built and tested in this study, we chose the latter as the first strategy. We used *Hind*III restriction sites at the 3′ end of the CP as the insertion site for constructing our first VIGS vector-pV1a23, a pXT1-CGMMV derivative missing the first restriction *Hind*III site at 5′ terminus and with the 159th amino acid of the CP mutated to a stop codon (Fig. 1a). Cucurbit plants inoculated with this vector showed viral symptoms on upper leaves similar to those of plants inoculated with the pXT1-CGMMV, and a progeny of CGMMV could be detected by DAS-ELISA and RT-PCR in these leaves (Fig. 1b, c; Additional file 1: Table S2).

**Fig. 2:**
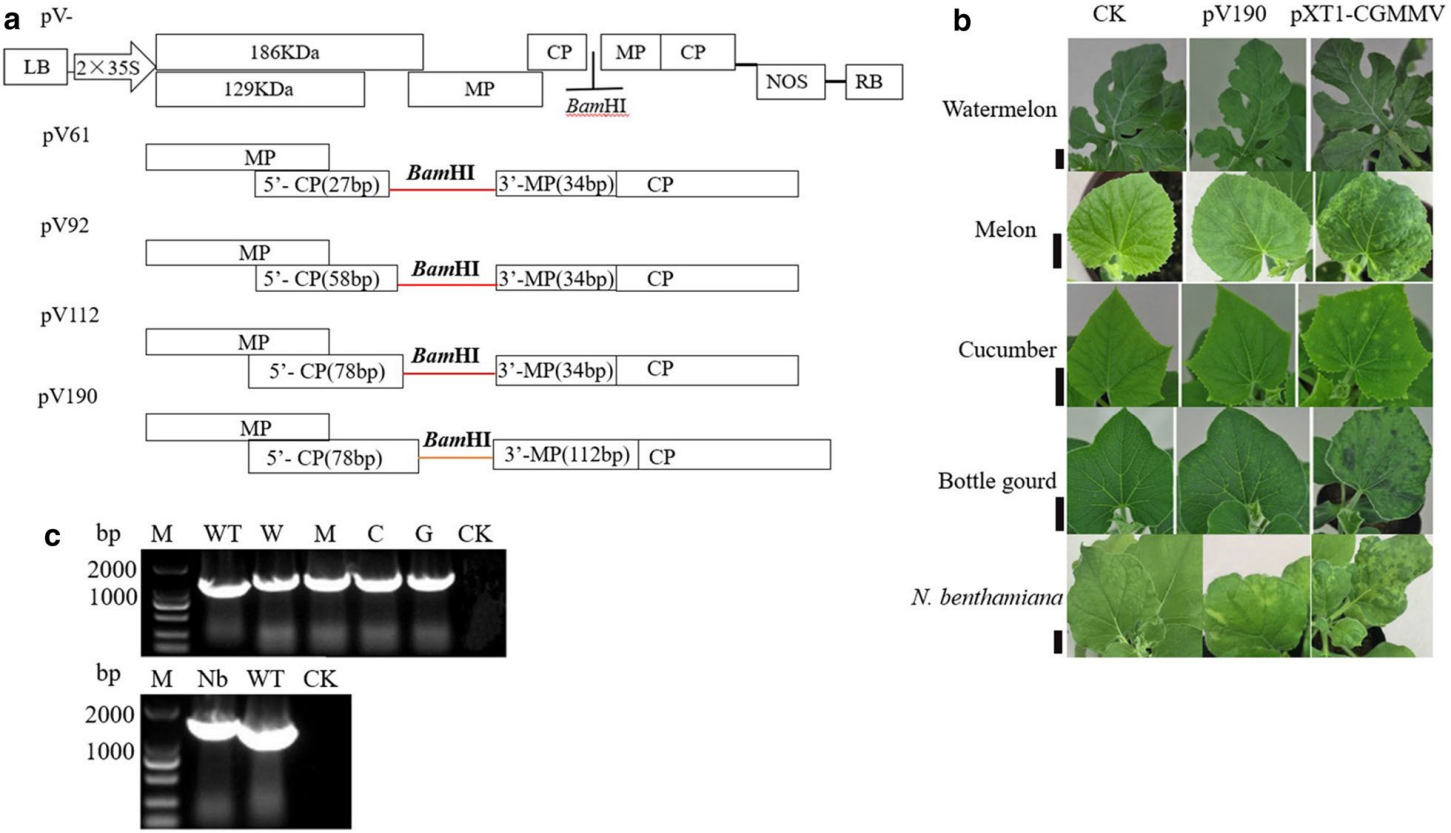
Engineering of CGMMV as a VIGS vector with different size CP subgenomic promoters. **a** Schematic representation of pV61, pV92, pV112 and pV190. pV- is a pXT1-CGMMV derivative that contains a direct repeat of the 61-, 92-, 112- and 190-bp putative CGMMV CP subgenomic promoter and a restriction enzyme site (*Bam*HI) between CP subgenomic promoters. **b** pV190 caused mild systemic symptoms on cucurbits and *N. benthamiana.* Bar = 1, 3, 3, 3 and 1 cm, respectively*.***c** RT-PCR detection of viral RNA showing that pXT1-CGMMV and pV190 are infectious in cucurbits and *N. benthamiana*. M, Marker2000; WT, wild type (pXT1-CGMMV); CK, negative control; W, M, C, G and Nb indicate watermelon, melon, cucumber, bottle gourd and *N. benthamiana*, respectively

Secondly, we determined the impact of a duplicated copy of the CP SGP in the viral genome on infections of VIGS [25], therefore we build vectors pV61, pV92 and pV112 (Fig. 2a). Viral symptoms could be observed on upper leaves of plants inoculated with vectors pV92 and pV112, whereas plants inoculated with pV61 did not develop viral symptoms and CGMMV could not be detected by DAS-ELISA. These results revealed that vectors pV92 and pV112 could infect plants systemically while pV61 could not.

Furthermore, our previous work revealed that the CP RNA transcription level was significantly enhanced when 105 nucleotides were retained before the CP transcription starting site (TSS) and that the sequence from the 71st base to the 91st base upstream of the CP TSS plays a key role in CP SGP activity [28]. Based on these results, we built pV190, a pXT1-CGMMV derivative that contains a direct repeat of the 190-bp putative CGMMV CP SGP and a single restriction site (*Bam*HI) between the duplicated CP SGPs (Fig. 2a). *N. benthamiana* and cucurbit plants inoculated with pV190 developed very mild symptoms on upper leaves, which were clearly milder than those of plants inoculated with the pXT1-CGMMV (Fig. 2b). However, the pV190 genomic RNA could be detected by RT-PCR (Fig. 2c), indicating that it could replicate and move systemically.

### Silencing effects of *PDS* fragments inserted in the sense orientation or conforming a hairpin

To determine whether pV1a23, pV92, pV112 and pV190 can be used to induce gene silencing in cucurbits, we chose to target *PDS* because it can result in striking photo-bleaching when silenced. Cucurbit *PDS* fragments with various lengths (114,150,213and300 bp) were amplified by selecting the region with the highest conservation. The sequence similarity of the 114-, 150- and 213-bp fragments in the four cucurbit species was approximately 97% (Additional file 2: Fig. S1a–c), While that of 300-bp fragments was 98.4%, but the fragment from watermelon contains an insertion of 30 bp (Additional file 2: Fig.S1d). The *PDS* fragments of 114-bp, 213-bp and 300-bp were inserted in the sense orientation at the *Hind*III cloning site of pV1a23 to produce pV1a23-PDS114, pV1a23-PDS213 and pV1a23-PDS300, respectively. Similarly, the *PDS* fragments of 150 bp and 213 bp were also inserted in the sense orientation at the *Bam*HI cloning site of pV92 and pV112 to produce pV92-PDS150, pV92-PDS213 and pV112-PDS150 and pV112-PDS213, respectively. The *PDS* fragments of 150 bp, 213 bp and 300 bp were inserted in the sense orientation at the *Bam*HI cloning site of pV190 to produce pV190-PDS150, pV190-PDS213 and pV190-PDS300, respectively.

**Fig. 3:**
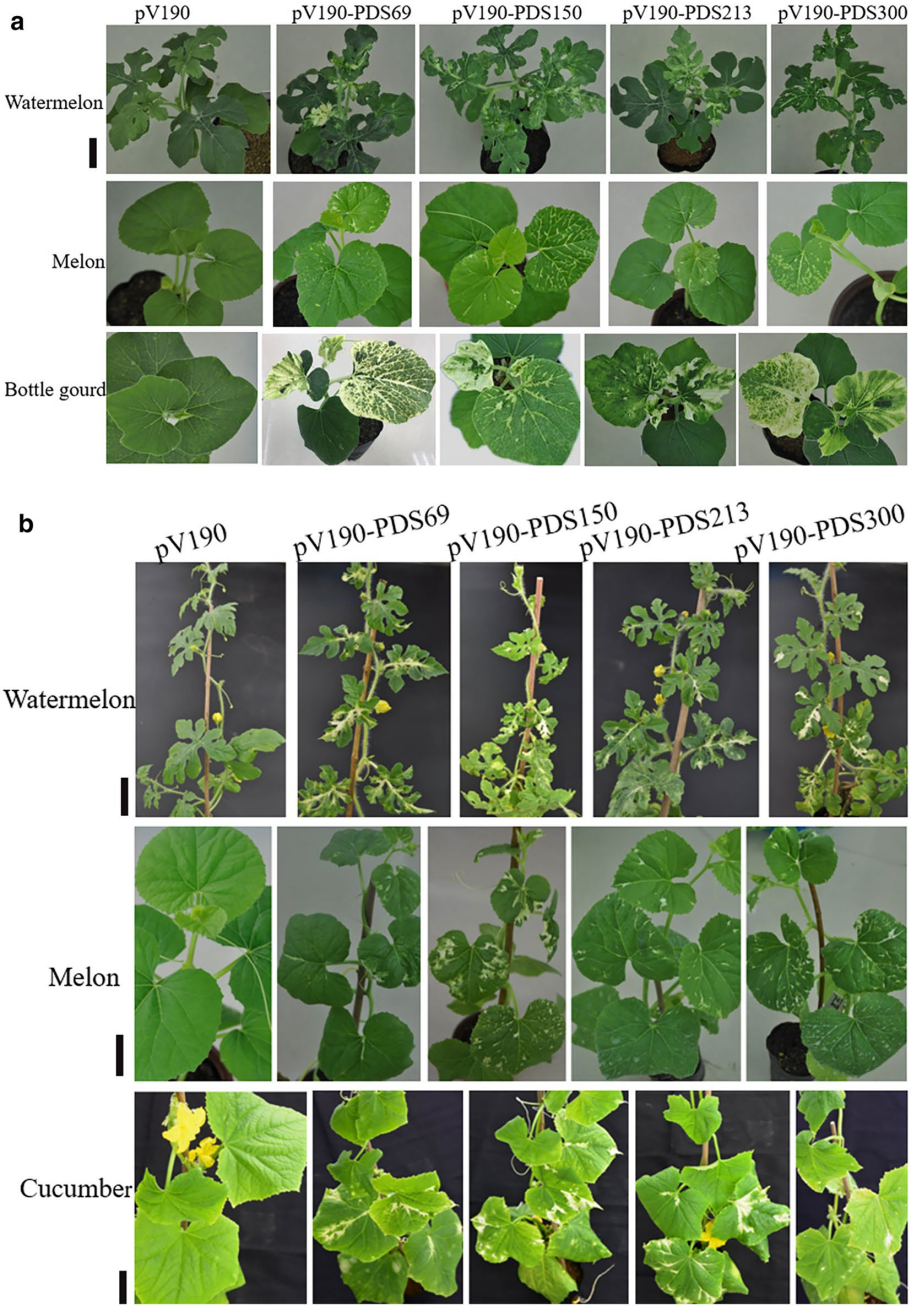
*PDS* silencing using the VIGS vectors pV190-PDS69, pV190-PDS150, pV190-PDS213 and pV190-PDS300. **a** Photobleaching was observed and photographed in watermelon at 19dpi, in melon at 12dpi and in bottle gourd plants at 22dpi. Bar = 3 cm. **b** Photobleaching was photographed in watermelon at 32 dpi, in melon at 20 dpi and in cucumber at 39 dpi, respectively. Bar = 7, 7 and 8 cm (from top to bottom)

**Fig. 4:**
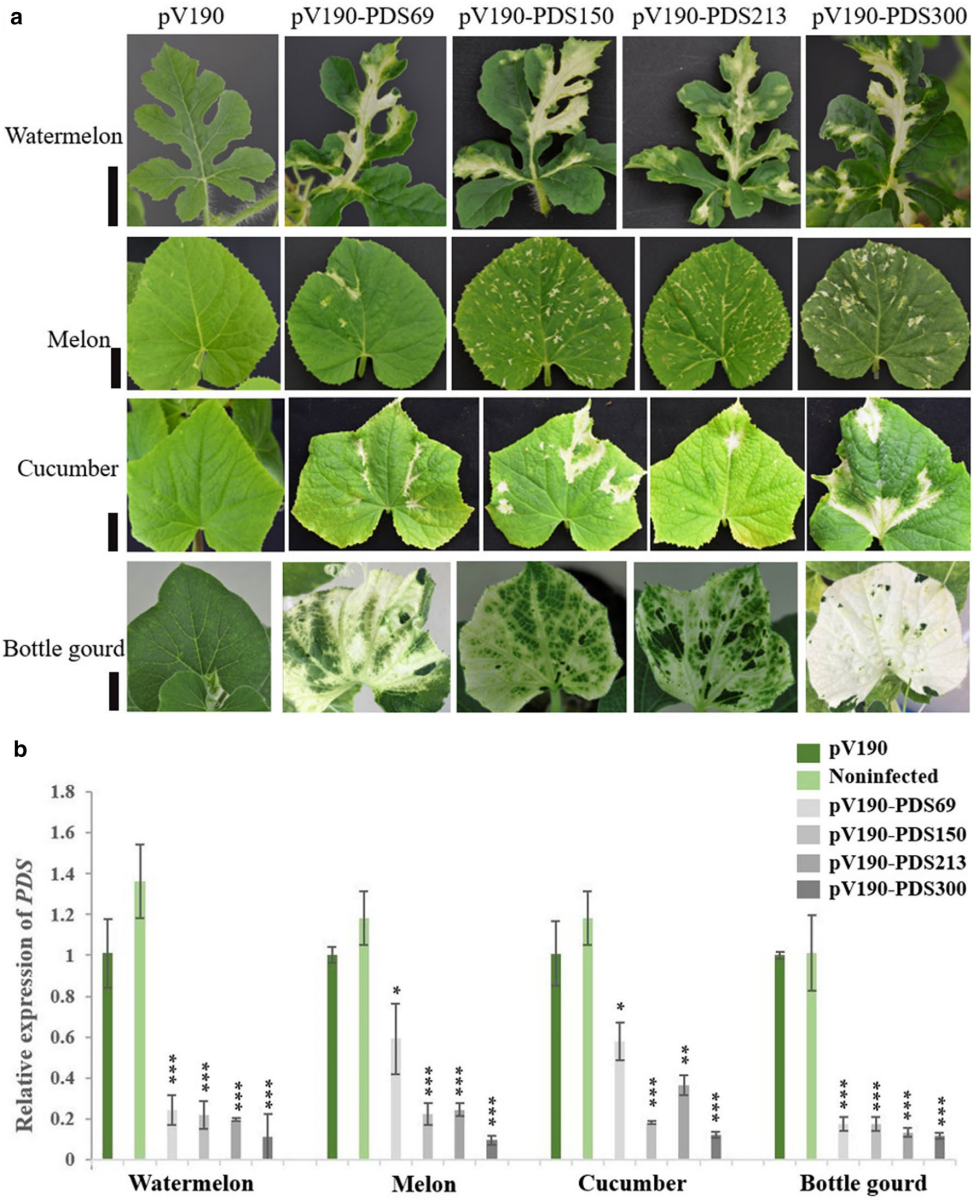
Silencing efficiency of VIGS vectors carrying *PDS* fragments of different sizes on cucurbits. **a** Indicate the uninoculated leaves displaying most obvious photobleaching on watermelon plants at 32 dpi, on melon at 27 dpi, on cucumber at 39 dpi and on bottle gourd at 34 dpi, respectively. Bar = 2, 3, 3 and 3 cm (from top to bottom). **b** Real-time qRT-PCR analysis of *PDS* expression in noninfected (NI), pV190 empty vector (EV), and CGMMV-PDS-infected cucurbit (watermelon, melon, cucumber and bottle gourd) plants. Three technical replicates were performed for each individual sample (*P < 0.05 and **P < 0.01 ***P < 0.001 compared with the empty vector (pV190) by Student’s t test. Error bars indicate the SD

To verify the silencing efficiency of these vectors, cucurbit plants (watermelon, melon, cucumber and bottle gourd) were subjected to *Agrobacterium*-mediated inoculation. Results revealed that pV1a23-PDS300 lost the ability of systemic and local infection, pV1a23-PDS213 was only able to infect locally and pV1a23-PDS114 could produce systemic and local infection (Additional file 1: Table S2), but failed inducing photobleaching (Fig. 1b), although the 114-bp *PDS* gene fragment was stable (Fig. 1c). Therefore, pV1a23 can not be used for VIGS. Photobleaching could be observed in the inoculated plants with pV92-PDS150 and pV112-PDS150 (Additional file 1: Table S2). However, pV92-PDS213- and pV112-PDS213-infected plants did not display any photobleached phenotype and the presence of CGMMV in the upper leaves was not observed (Additional file 1: Table S2). The cotyledons of watermelon, melon, cucumber and bottle gourd seedlings were inoculated with pV190-PDS150, pV190-PDS213 and pV190-PDS300. Photobleaching was first observed among all plants inoculated with different vectors on the 4th true leaves (L4) in watermelon at about 19 dpi, on the 3rd leaves (L3) in melon and bottle gourd at 12 dpi (Fig. 3a), and on the 5th true leaves of cucumber at 28 dpi. Further, photobleaching was observed up to 32, 20 and 39 dpi in watermelon, melon and cucumber plants, respectively (Fig. 3b). About 70% of the inoculated plants showed a photobleaching phenotype. Total RNA was extracted from leaves of the plants inoculated with different vectors displaying the most obvious photobleaching (Fig. 4a) and the accumulation of *PDS* transcripts was quantified by qRT-PCR. The results showed that the expression levels of *PDS* had no significant differences between pV190-infected (Empty Vector, EV) and noninfected (NI) leaves, demonstrating that pV190 did not significantly affect *PDS* expression (Fig. 4b). The *PDS* mRNA transcript levels in photobleached leaves was reduced by approximately 79%, 81% and 89% in watermelon, 78%, 76% and 81% in melon, 83%, 87% and 89% in bottle gourd, and 82%, 64% and 88% in cucumber infected with pV190-PDS150, pV190-PDS213 and pV190-PDS300, respectively, compared to plants infected with pV190 (Fig. 4b).

Further, to improve silencing efficiency, we inserted a *PDS* fragment forming a hairpin structure of 69-bp into pV190 to produce the pV190-PDS69 vector. We first observed photobleaching phenotype among all plants inoculated with different vectors on the L5 in watermelon at 17 dpi and photographed at 19dpi, on the L2 in melon at 10 dpi and at 12 dpi, on the L3 in bottle gourd at 11 dpi and at 22 dpi (Fig. 3). Photobleaching was first observed one or two days earlier in plants infected with pV190-PDS69 than in plants infected with any other vector. The *PDS* mRNA levels declined 76%, 41%, 42% and 83% in watermelon, melon, cucumber and bottle gourd, respectively (Fig. 4b).

### Stability of the 69–300-bp *PDS* fragments in pV190

**Fig. 5:**
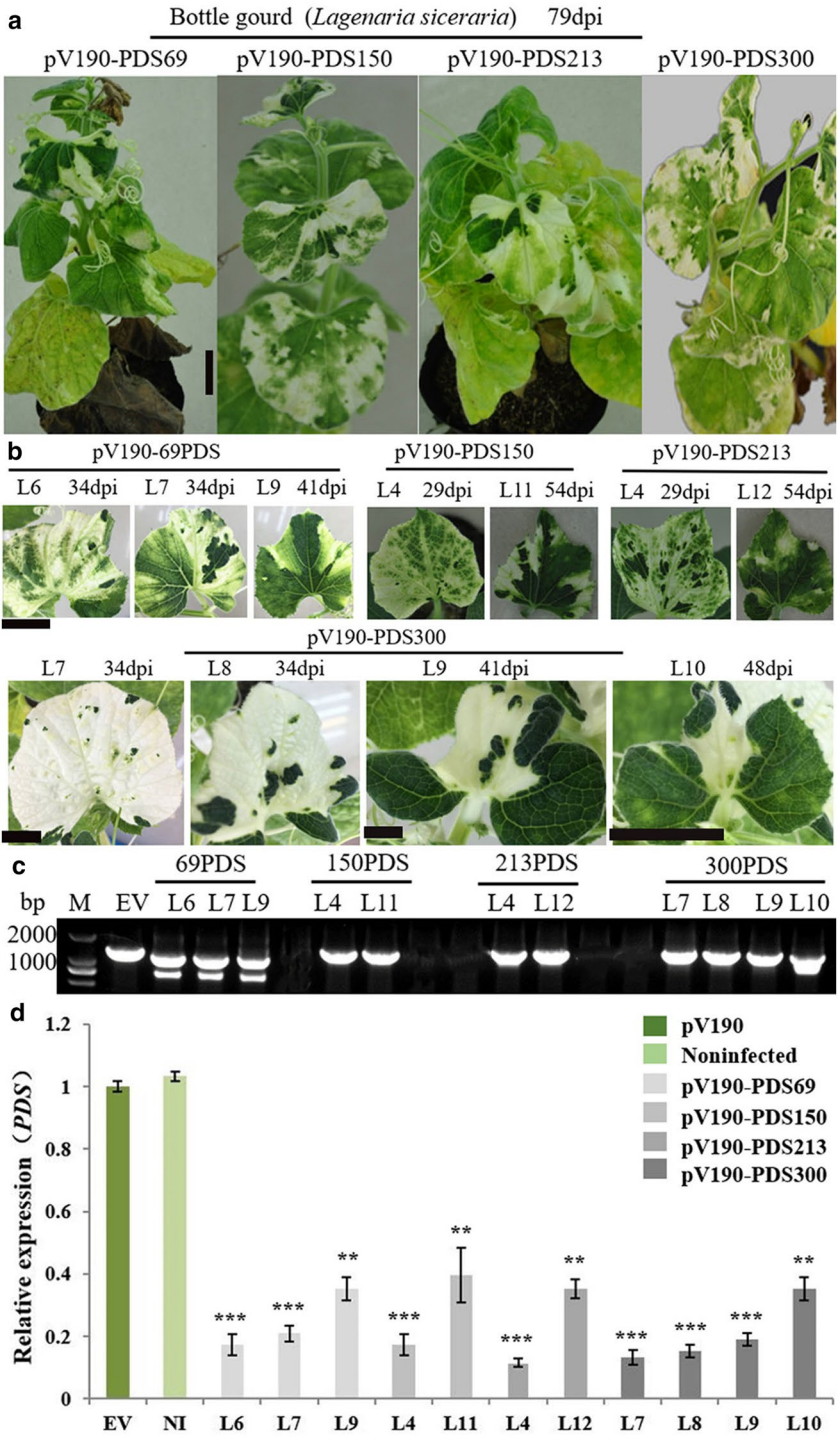
Silencing efficiency and stability of the pV190 VIGS vector with different length inserts in bottle gourd. Fragments of 69 bp (dsRNA hairpin structure), 150 bp, 213 bp, 300 bp were separately cloned into pV190. **a** Silencing *PDS* using pV190 on bottle gourd plants produced photobleaching that persisted for over 70 days. Bar = 4 cm, **b** Photobleaching on newly emerging leaves of bottle gourd plants caused by *PDS* silencing was observed at 29, 34, 41, 48 and 54 dpi, respectively. Bar = 2, 1, 2 and 2 cm. **c** RT-PCR assay to detect the presence of pV190 carrying *PDS* fragments of different sizes in systemic leaves. Samples from the 4rd leaf above the inoculated (L4) were collected at 29 dpi; L6, L7 and L8 samples were collected at 34 dpi, L9 sample was collected at 41 dpi, L10 at 48 dpi, L11 and L12 at 54 dpi. M: Marker2000; EV: Empty vector (pV190). **d** Relative expression level of *PDS* mRNA in the above indicated leaves determined by real-time qRT-PCR

We observed that the silencing phenotype of the *PDS* gene could persist for over 2 months in bottle gourd (Fig. 5a). Photobleaching was not uniform from bottom to top of bottle gourd leaves (Fig. 5b). To evaluate the stability of the *PDS* fragment in CGMMV-vectors, RT-PCR was performed on total RNA extracted from bottle gourd leaves L6, L7 and L9 for pV190-PDS69, L4 and L11 for pV190-PDS300, L4 and L12 for pV190-PDS213, and L7, L9 and L10 for pV190-PDS300 (Fig. 5b). The result showed the 150-bp and 213-bp *PDS* fragments were stable across all analyzed leaves. The 69-bp dsRNA hairpin structure could not be detected across all leaves, whereas L9 and L10 samples from pV190-PDS300-infected bottle gourd contained deletions of the 300-bp *PDS* fragment to different extents (the deletion in L9 was less than the L10) (Fig. 5c). The relative expression of the *PDS* gene in the above same leaves was measured by qRT-PCR. RT-PCR results corresponded well with the *PDS* relative expression level measured by qRT-PCR, with less silencing observed as the extent of deletions increased (Fig. 5c, d). For instance, pV190-PDS69 caused *PDS* transcripts to be reduced by 83%, 80% and 65% in the L6, L7, L9, respectively, and pV190-PDS300 caused *PDS* transcripts to be reduced by 87%, 81% 73% and 65% in the L7, L8, L9 and L10, respectively (Fig. 5d). Results of stability of the 69–300-bp *PDS* fragments in pV190 in watermelon, melon and cucumber were consistent with those in bottle gourd. The expression of *PDS* in the youngest analyzed leaves was still down-regulated (Fig. 5d), indicating that these vectors have sufficient stability to be used to characterize gene functions in cucurbit plants.

### The silencing effect of CGMMV-based vectors could be passaged

**Fig. 6:**
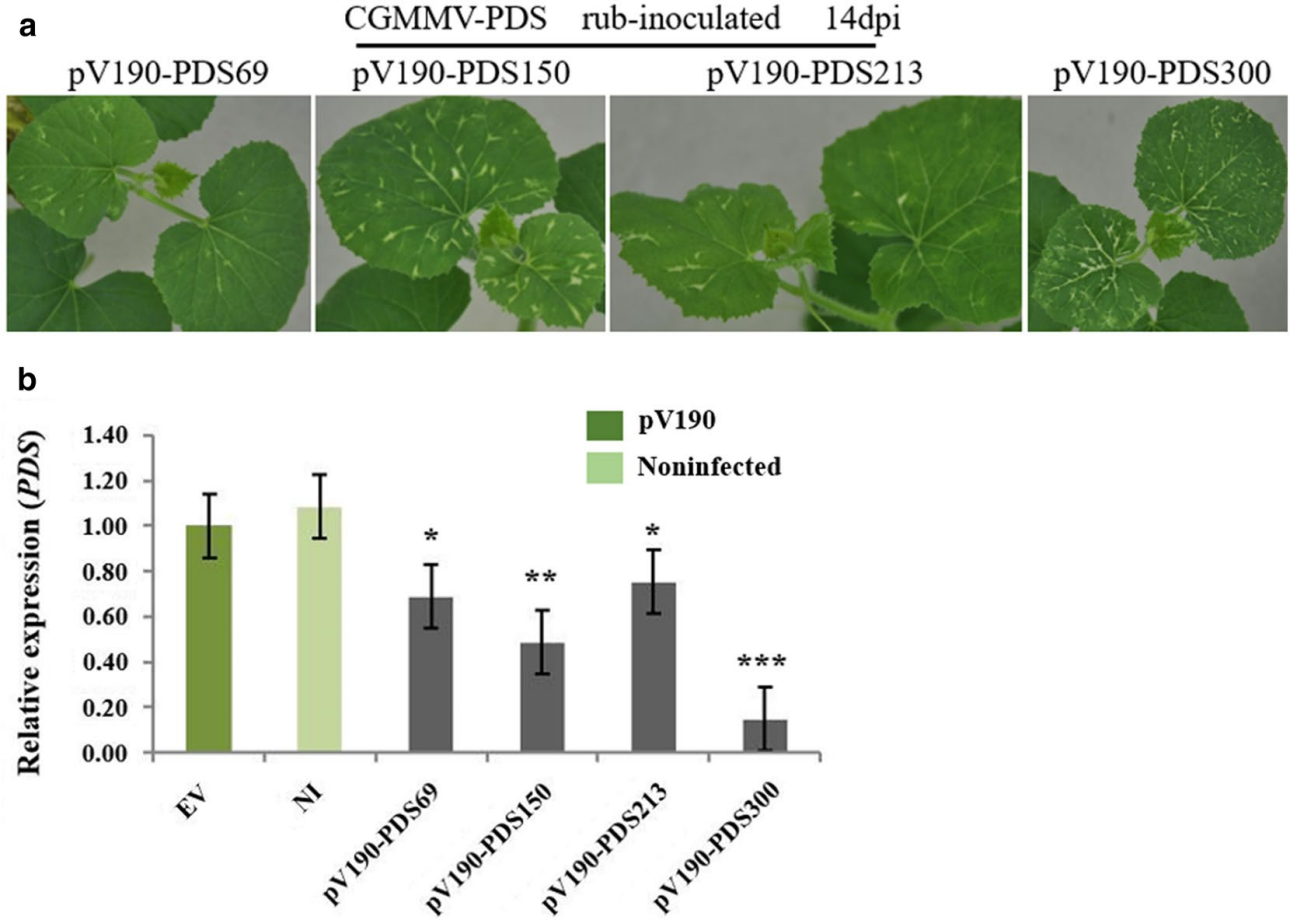
The silencing effect of pV190-PDS69, -PDS150, -PDS213 and -PDS300 could be passaged. **a** Photobleaching caused by *PDS* silencing in systemic leaves of melon plants that were rub inoculated with sap from pV190-PDS69, -PDS150, -PDS213 and -PDS300-infected leaf tissue. The photobleaching phenotype was observed and photographed at 9dpi/14dpi. **b** Real-time qRT-PCR analysis of *PDS* expression in the 5th leaf above the inoculated (L5) of noninfected (NI), pV190 empty vector (EV), and pV190-PDS69, -PDS150, -PDS213 and -PDS300-infected melon by mechanical inoculation

To verify whether the silencing vectors can be passaged, the sap of leaves with obvious photobleaching was used to rub-inoculate cotyledons and the L1 of melon plants. Photobleaching occurred on uninoculated leaves as early as 9 dpi and was photographed at 14 dpi (Fig. 6a). *PDS* expression levels were tested on L5 of passaged plants. We observed that *PDS* relative expression was reduced by 32%, 52%, 25% and 85% in pV190-PDS69, -PDS150, -PDS213 and -PDS300, respectively (Fig. 6b), confirming that the silencing effect of CGMMV-based vectors could be passaged.

### CGMMV-based VIGS in *N. benthamiana*

*Nicotiana benthamiana* is an important experimental host for CGMMV. We utilized two different lengths of *PDS* fragments which were amplified by selecting conserved regions of *PDS* gene sequences in *N. benthamiana* to test whether CGMMV is competent to induce gene silencing in *N. benthamiana* plants. At 14 dpi, a little weak photobleaching could be observed in the upper leaves of all plants inoculated with either pV190-NbPDS146 or pV190-NbPDS215 (Additional file 3: Fig. S2a). Consistently, qRT-PCR results showed that the expression of *PDS* in pV190-NbPDS146- and pV190-NbPDS215-infected leaves was reduced by 60% and 34%, respectively, compared with the pV190 infected leaves (Additional file 3: Fig. S2b).

## Discussion

In this study, we successfully developed a new CGMMV-based VIGS vector which could be used to silence endogenous genes in cucurbit plants. Using this viral system, we successfully silenced *PDS* in cucurbits including watermelon, melon, cucumber and bottle gourd and in the model plant *N. benthamiana*. To our best knowledge, this is the first time that CGMMV has been engineered as a VIGS vector, although it has been exploited for protein overexpression.

During the process of modifying the CGMMV genome to produce a VIGS vector, we observed that the insertion sites of the gene fragment determined the viability, stability, insert size and silencing efficiency of the vector; our work showed that a duplicated copy of the 190-bp putative CGMMV CP SGP was essential for silencing. We first tried to place the foreign gene insertion site downstream of the viral CP gene. Results demonstrated that the insertion site between the CGMMV CP gene stop codon and the 3‵ non-coding region was not suitable for constructing the VIGS vector. TMV is a member of the genus *Tobamovirus* and has successfully been developed as a VIGS vector by utilizing the strategy of subgenomic expression [25]. Because CGMMV is also a member of the genus *Tobamovirus* and CGMMV infectious clone containing the green fluorescence protein (GFP) reporter gene has been successfully constructed, the GFP gene was located in between MP and CP [27]*.* Thus, we adopted a similar strategy as reported for TMV to generate a CGMMV VIGS vector and explored different lengths of the duplicated region. For the impact of length of 61-, 92-, 112- and 190-bp duplicated copy of the putative CGMMV CP SGP on silencing, only when the modified CGMMV-based vector contained a 190-bp duplicated copy of the putative CGMMV CP SGP, the *PDS* gene fragments could induce a robust silencing phenotype. These results suggest that it is necessary to create an additional fully competent subgenomic promoter to drive the transcription of the VIGS target sequence and for providing the vector with the ability to systemically infect plants [29].

Vectors containing duplicated sequences frequently suffer partial or complete loss of inserted sequences, particularly when the insert size is large [30, 31]. We tested the effect of the length and structure of inserts on silencing. Our results showed that the CGMMV vectors harboring the sense-oriented *PDS* gene sequence of 100–300 bp in length could effectively induce silencing in cucurbits, and efficiency was highest for the largest fragment, the 300-bp *PDS* gene fragment. It is worth mentioning the effect on silencing of the cDNA insert length in a tobacco rattle virus (TRV)-based vector [32]. The better silencing phenotype could be produced when the cDNA insert length was between 200 and 1300 bp, whereas inserts shorter than 190 bp and longer than 1661 bp generated less siRNAs silencing less efficiently [32]. Not only the length of the insert affected silencing but also the structure of it has an impact on silencing. Expression of a hairpin-loop dsRNA structure could enhance the efficiency of VIGS [33]. This seems to be true for our CGMMV-based VIGS vector. The silencing efficiency of a 69-bp hairpin-loop structure was between that of the 150-bp and 300-bp sense constructs, but its silence phenotype appeared earlier. A direct 60-bp inverted-repeat sequence of the target gene that could fold as dsRNA strongly enhanced VIGS from foxtail mosaic virus (FoMV) [16]. However, in our work, a 60-bp inverted-repeat sequences of the *PDS* gene could not produce photobleaching, and CGMMV lost the ability of systemic infection (unpublished data). Therefore, these results suggest that the effect of length and structure of inserts on silencing varied with vectors from different viruses.

Furthermore, the stability of inserts in the pV190 vector were evaluated. The photobleaching phenotype was observed from the 3rd to the 11th leaves and *PDS* transcripts were reduced by about 80% and 20% in L4/L5 and L10/L11. About 70% of tested plants had a photobleaching phenotype which was stable and persisted for over 2 months. Stable photobleaching was also observed in plants mechanically inoculated with leaf sap prepared from L5/L6 of CGMMV-PDS inoculated plants. Here, it is worth mentioning that *PDS* transcript abundance could be reduced by CGMMV-PDS vectors on the 3rd to 10th leaves of the tested plants. However, the photobleaching phenotype and the *PDS* transcript levels were not uniform in these leaves, and could produce a gradient from bottom to top. It has been reported that the phenomenon could be due to instability of the *PDS* gene fragment. RT-PCR analyses on all of these leaves showed that deletion of the *PDS* insert was hardly detected in samples with the sense inserts. Hence, we reasoned that gene-silencing efficiency may be related to the accumulation of the foreign fragment-derived siRNAs [34, 35], but the specific mechanism of action remains unclear. In contrast, the full-length sequence of the insert in the pV190-PDS69 vector could not be detected in photobleached leaves. This phenomenon may be explained by a systemic silencing signal that can be actively transmitted over long distances through the phloem to induce *PDS* gene silencing in young leaves [35–37], but the exact molecular form of a mobile RNA signal in the phloem still needs further research.

In addition, we tested whether pV190 can be used to induce gene silencing in *N. benthamiana*. Results showed that pV190 could infect *N. benthamiana* leaves, the uninoculated systemic leaves developed very mild symptoms. The pV190 vectors, harboring 146-bp and 215-bp *PDS* fragments could trigger silencing, but the photobleaching phenotype was not striking. The photobleaching phenotype also varied in cucurbit plants, with the most obvious phenotype in bottle gourd. We reasoned that the viral vector has different fitness for different hosts. The accumulation of siRNAs is a crucial factor for silencing efficiency, while the host species also contain crucial factors including the *DCL, RDR* and *AGO* genes [38–40].

Apple latent spherical virus was first described as a vector for gene silencing in cucurbits [11], and few additional studies have reported its application for VIGS in cucurbit plants. A new TRSV vector was recently reported [17]. More recently, the TRV-VIGS system has been used in cucumber and oriental melon [18, 19]. Comparing ALSV, TRSV and TRV with CGMMV, the first three belong to multipartite virus families with a bipartite genome, while CGMMV is a monopartite virus [22], and therefore is easier to manipulate. The ALSV genome is expressed through polyprotein synthesis followed by proteolytic processing, which represents another layer of difficulty for high throughput functional genomics [41, 42]. A second major difference among the four cucurbit viral vectors is the inoculation method. *A. tumefaciens* infiltration is a simple, effective and convenient inoculation way [43, 44] and TRSV, TRV and CGMMV vectors are designed for agroinfiltration [17, 45]. However, ALSV does not adopt this inoculation method. Host range is another major difference. TRSV cDNA clones are not infectious in watermelon or pumpkin [17]. Although TRV has been widely used as VIGS vector since it has a wide host range [46], its application for VIGS on cucurbits except cucumber and oriental melon have not been reported [18, 19]. Both ALSV and CGMMV vectors can be successfully used on common cucurbit plants such as watermelon, melon, cucumber and bottle gourd [11]. In short, the CGMMV-based VIGS vector pV190 is easy to handle during either the process of preparing a VIGS construct or for inoculations. Besides, it can systemically infect *N. benthamiana* and various cucurbit species and produce very mild viral symptoms in upper leaves of inoculated plants. Silencing phenotypes caused by pV190-based VIGS vectors were stable and could persist for at least one month.

## Conclusions

CGMMV has a broad host range including 29 species, of which at least 16 belong to *Cucurbitaceae* [20]. Thus, the pV190 VIGS vector should have the potential for VIGS in many other plants although we only evaluated its application in *N. benthamiana*, watermelon, melon, cucumber and bottle gourd. Taken together, the CGMMV-based silencing system could be applied as a powerful biotechnological tool with a great potential for studying functional genomics in cucurbits. Future study will focus on obtaining insights into the molecular mechanism underlying the difference in silencing efficiency between different plants. In addition, the vector could serve as a basis to control devastating viral pathogens or carry out genetic engineering and molecular breeding.

## Materials and methods

### Plant materials

The CGMMV experimental host *N. benthamiana* and cucurbits hosts (watermelon, melon, cucumber and bottle gourd) were used for VIGS of the *PDS* gene by CGMMV vectors in this study. Watermelon (Zhengkang 2), melon (Baimei), cucumber (Jinyan 4) and bottle gourd (Yongzhen1) seeds were obtained from Zhengzhou Fruit Research Institute (Zhengzhou, China), Xinjiang Academy of Agricultural Sciences (Xinjiang, China), Tianjin Academy of Agricultural Sciences (Tianjin, China) and Ningbo Academy of Agricultural Sciences (Ningbo, China), respectively. All cucurbit seeds were soaked in sterile water for 3–4 h at 50 °C, then placed in Petri plates containing wetted filter cotton gauze at 28 °C in darkness until seeds were germinated. Germinated seeds were planted into pots with nutrient matrix and grown in a growth chamber under 16 h light at 28 °C/8 h dark at approximately 22 °C. The same conditions were used to grow inoculated plants (see below) with CGMMV vectors.

## Construction of the CGMMV-based vectors

pV1a23 was constructed by site-directed mutagenic PCR using primer pairs Del*Hind*III-X/ S159Z-S and S159Z-X/Del*Hind*III-S (Additional file 4: Table S1). To construct pV61, pV92, pV112 and pV190 VIGS vectors, pXT1-CGMMV was modified by removing the CP start codon PCR using primer pairs CP-TC-F and CP-TC-R (Additional file 4: Table S1), resulting in the single-nucleotide substitution ATG to ACG. The resulting construct was named pXT1-CGACG. Further, DNA fragment 1 containing CGMMV nt 1-(5711–5840) (GenBank accession: KY753929) was amplified using pXT1-CGMMV as a template with primer pairs PXT1-F/(27B-34-R, 58B-34-R, 78B-34-R or 78B-99-R), whereas DNA fragment 2 containing CGMMV nt 5651/5716–6423 was amplified using pXT1-CGACG as a template with primer pairs PXT1-R /(27B-34-F, 58B-34-F, 78B-34-F or 78B-99-F) (Additional file 4: Table S1). These two fragments were ligated by homologous recombination. The resulting vectors pV61, pV92, pV112 and pV190 are pXT1-CGMMV derivatives that include a duplicated copy of 61-bp, 92-bp, 112-bp and 190-bp putative CGMMV CP SGP respectively and a single restriction site (*Bam*HI) between the duplicated CP SGP.

### Insertion of different *PDS* fragments into the CGMMV-based vector

For a VIGS test with *PDS* as the target gene, a series of pV1a23, pV92, pV112 and pV190-based vectors harboring different *PDS* fragments of varied sizes were constructed. Twelve primer sets CuPDS-*Hind*III-F/R, CuPDS-*Hind*III-2F/2R, CuPDS-*Hind*III-3F/3R, 58–150-F/R, 78–34-150F/R, 78–150-F/R, 58–213-F/-R, 78–34-213F/R, 78–213-F/R, 78–300-F/R, 78–146 N-F/R and 78–215 N-F/R were designed to amplify 114-, 150-, 213-, and 300-bp fragments of the cucurbit *PDS* gene and 146-, 215-bp fragments of the *N. benthamiana PDS* gene, respectively (Additional file 4: Table S1). These resulting fragments were inserted into digested pV1a23 with *Hind*III and pV92, pV112, pV190 with *Bam*HI in sense orientation by homologous recombination, respectively. The resulting three pV1a23-derived and ten pV92, pV112 and pV190-derived constructs were named pV1a23-PDS114, pV1a23-PDS213, pV1a23-PDS300, pV92-PDS150, pV92-PDS213, pV112-PDS150, pV112-PDS213, pV190-PDS150, pV190-PDS213, pV190-PDS300, pV190-NbPDS146 and pV190-NbPDS215. pV190-PDS69, a construct carrying a 69-bp fragment (dsRNA hairpin structure) of the cucurbit *PDS* gene, was amplified using pV190 as template and three primers sets 78-69P-X/CG-4R, CG-4F/78-69P-S and 3R/ TxR ~ R, respectively (Additional file 4: Table S1).

### Agroinfiltration and sap inoculation

All constructs were introduced into *Agrobacterium tumefaciens* strain GV3101 by freeze–thaw transformation, then single clones were picked up and transferred into 200 µL LB liquid media containing kanamycin (50 µg mL^−1^) and rifampicin (50 µg mL^−1^) and cultured overnight in a shaker at 28 ℃. The bacterium culture was mixed with LB at a 1:100 ratio and cultured in a shaker overnight, followed by centrifugation at 6000*g* for 5 min to collect the bacteria. The bacteria were resuspended in inducing buffer solution containing 10 mmol L^−1^ MgCl_2_, 10 mmol L^−1^ MES, and 100 µmol L^−1^ Acetosyringone, and the final OD_600_ value was adjusted to 0.8–1. The cells were maintained at room temperature (25 ℃) for at least 2 h before agroinoculation. The upper 2–3 leaves of *N. benthamiana* at the 6–8 leaf stage and cotyledons from 14-day-old cucurbit seedlings were infiltrated with the *A. tumefaciens* suspension using a 1-mL syringe.

In order to verify whether the silencing effect of these vectors could be passaged, the sap from leaves of the agroinfiltrated melon plants displaying obvious photobleaching was used to rub-inoculate cotyledons and the first true leaf (L1) of the melon plants. Each experiment was repeated at least three times, with 9 plants for each construct in each experiment.

### DAS-ELISA and RT-PCR

After agroinfiltration and sap inoculation, CGMMV in inoculated plants was detected by DAS-ELISA and RT-PCR at specific time points. DAS-ELISA was performed to detect CGMMV accumulation using an ELISA kit (Adgen, Auchincruive, UK). For RT-PCR, total RNA was extracted from cucurbit (watermelon, melon, cucumber and bottle gourd) and *N. benthamiana* leaf tissues using the RNA simple kit (Tiangen Biotech, Beijing, China) and then first-strand cDNA was synthesized from 1 μg total RNA using an oligo dT primer according to the protocol of PrimescriptII RT (TAKARA). PCR was performed with primer set 5574F and 3UTR that flanked the foreign insert to detect CGMMV and asses the stability of the pV190 and foreign inserts of CGMMV-based vectors (Additional file 4: Table S1).

### qRT-PCR analysis

qRT-PCR was performed to measure the mRNA expression level of the endogenous *PDS* genes using the SYBR Green I Master (Roche) in either *N. benthamiana* or cucurbit plants inoculated with CGMMV-based vectors at specific time points. The first-strand cDNA was synthesized from 1 μg total RNA using an oligo dT primer according to the protocol of PrimeScript™RT reagent Kit with gDNA Eraser (TAKARA). The expression level of *PDS* of cucumber, melon, gourd and watermelon was determined using primer sets CuPDS-679F/CuPDS-906R and wate-q-F/R, respectively, designed to prime outside the region targeted for silencing (Additional file 4: Table S1). Expression of the actin gene by primer set cumsactin-F/R (Additional file 4: Table S1) was used as an internal control of cucumber, melon, gourd plants. The *ClCAC* gene was used as an internal control of watermelon plants using primer pairs Cla016178-F/R [47]. The primer set NbPDS-qF/R was designed for detecting the expression of *PDS* in *N. benthamiana*, and the expression of the GAPDH gene analyzed by the primer set GAPDH-qRT-F/R was referred as an internal control (Additional file 4: Table S1). The expression *PDS* was calculated using the 2^−∆∆CT^ method [48]. The expression level of *PDS* in the negative control (pV190) was set to an arbitrary value (1.0) to calculate the relative expression levels of the other samples, with 3 replicates used for each sample.

### Accession numbers

Sequence data from this article can be found in the GenBank or Cucurbit Genomics Database (https://cucurbitgenomics.org/) under the following accession numbers: CGMMV (KC851866); *Nicotiana benthamiana PDS* (EU165355); *Cucumis sativus PDS* (XM_011654729); *Cucumis melo PDS* (NM_001297530); *Citrullus lanatu PDS* (Cla010898; ClCG07G015130); *Lagenaria siceraria PDS* (Lsi07G003470).

## Supplementary information

**Additional file 1: Table S2.** The infection analysis of pV1a23 (insertion sites behind the viral CP gene) vector and modified CGMMV-based vector containing a duplicated copy of the 61-, 92-, 112- and 190-bp putative CGMMV CP SGP.

**Additional file 2: Fig. S1.** The sequence similarities of 114-, 150- , 213- and 300-bp PDS gene fragments in four cucurbit species. a, b, c and d correspond to *PDS* fragments of 114-, 150- , 213- and 300-bp, respectively, in the four cucurbit species. HG, HUG, XG and TG represented cucumber, bottle gourd, watermelon and melon, respectively.

**Additional file 3: Fig. S2.** Silencing efficiency of different length inserts (*PDS*) using the pV190 VIGS vector in *N. benthamiana*. Fragments of 146 bp, 215 bp were separately cloned into pV190 VIGS vector. (a) The silencing phenotypes were observed at 14dpi. Bar = 1 cm. (b) The relative expression level of *PDS* mRNA determined by real-time qRT-PCR.

**Additional file 4: Table S1.** Primers used in this study.

## Data Availability

All data generated or analyzed during this study are available in this published article.
