# Supplementary material for: A cucumber green mottle mosaic virus vector for virus-induced gene silencing in cucurbit plants
Source: Plant Methods. 2020 Feb 3;16:9. doi: 10.1186/s13007-020-0560-3 (PMC6996188; doi:10.1186/s13007-020-0560-3)
Supplement: Supplementary file 1 — Additional file 1: Table S2. The infection analysis of pV1a23 (insertion sites behind the viral CP gene) vector and modified CGMMV-based vector containing a duplicated copy of the 61-, 92-, 112- and 190-bp putative CGMMV CP SGP. [file 13007_2020_560_MOESM1_ESM.doc]

**Table S2.** The infection analysis of pV1a23 (insertion sites behind the viral CP gene)

**vector and modified CGMMV-based vector containing a duplicated copy of the 61-, 92-, 112- and 190-bp putative CGMMV CP SGP.**

| **Vectors** |  | **14dpi** | | **21dpi** | |
| --- | --- | --- | --- | --- | --- |
|  |  | **Symptom** | **ELISA** | **Symptom** | **ELISA** |
| **pV1a23** | **IL** |  | **+** |  | **+** |
|  | **SL** | **M** | **+** | **M** | **+** |
| **pV1a23-PDS114** | **IL** |  | **+** |  | **+** |
|  | **SL** | **M^-^** | **+** | **M^-^** | **+** |
| **pV1a23-PDS213** | **IL** |  | **+** |  | **+** |
|  | **SL** | **-** | **-** |  | **-** |
| **pV1a23-PDS300** | **IL** |  | **-** |  | **-** |
|  | **SL** | **-** | **-** |  | **-** |
| **WT** | **IL** |  | **+** |  | **+** |
|  | **SL** | **M** | **+** | **M** | **+** |
| **CK** | **IL** |  | **-** |  | **-** |
| **pV61**  **pV92**  **pV92-PDS150**  **pV92-PDS213**  **pV112-PDS150**  **pV112-PDS213**  **pV190-PDS150**  **pV190-PDS213**  **pV190-PDS300** | **SL**  **IL**  **SL**  **IL**  **SL**  **IL**  **SL**  **IL**  **SL**  **IL**  **SL**  **IL**  **SL**  **IL**  **SL**  **IL**  **SL**  **IL**  **SL** | **-**  **M**  **W**  **W**  **W**  **W**  **W** | **-**  **+**  **-**  **+**  **+**  **+**  **+**  **+**  **-**  **+**  **+**  **+**  **-**  **+**  **+**  **+**  **+**  **+**  **+** | **M**  **W**  **W**  **W**  **W**  **W** | **-**  **+**  **-**  **+**  **+**  **+**  **+**  **+**  **-**  **+**  **+**  **+**  **-**  **+**  **+**  **+**  **+**  **+**  **+** |

pV1a23 is a pXT1-CGMMV derivative that contains a restriction enzyme site (*Hin*dIII) behind the viral CP gene. pV1a23-PDS114, pV1a23-PDS213and pV1a23-PDS114, is pV1a23 vector carrying three different *PDS* fragment (114 bp, 213 bp and 300 bp). pV61, pV92, pV112 and pV190 are pXT1-CGMMV derivative that contains a direct repeat of the 61-, 92-, 112- and 190-bp putative CGMMV CP subgenomic promoter and a restriction enzyme site (*Bam*HI) between CP subgenomic promoters. WT: wild type (pXT1-CGMMV). CK: blank control check. “IL” and “SL” indicate inoculated leaves and systemic infection leaves, respectively. “M” and “M^-^” indicate symptom of mosaic and mild mosaic, respectively. “W” indicates photobleaching phenotype on newly emerging leaves caused by *PDS* silencing. “+” and “-”, positive and negative in DAS-ELISA, respectively.
