# Supplementary material for: A cucumber green mottle mosaic virus vector for virus-induced gene silencing in cucurbit plants
Source: Plant Methods. 2020 Feb 3;16:9. doi: 10.1186/s13007-020-0560-3 (PMC6996188; doi:10.1186/s13007-020-0560-3)
Supplement: Supplementary file 2 — Additional file 2: Fig. S1. The sequence similarities of 114-, 150- , 213- and 300-bp PDS gene fragments in four cucurbit species. a, b, c and d correspond to PDS fragments of 114-, 150- , 213- and 300-bp, respectively, in the four cucurbit species. HG, HUG, XG and TG represented cucumber, bottle gourd, watermelon and melon, respectively. [file 13007_2020_560_MOESM2_ESM.pdf]

(a)

```
CLUSTAL X (1.83) multiple sequence alignment


HG              ATGCTTACTTGGCCAGAGAAAATTAAATTTGCAATTGGGCTCCTGCCGGCAATGCTTGGT
HUG             ATGCTTACTTGGCCAGAGAAAATTAAATTTGCAATTGGGCTCCTGCCGGCAATGCTTGGT
XG              ATGCTTACTTGGCCTGAGAAAATTAAATTTGCAATTGGGCTCCTGCCAGCAATGCTTGGT
TG              ATGCTTACTTGGCCAGAGAAAATTAAATTTGCAATTGGGCTCCTGCCAGCAATGCTTGGT
                ************** ******************************** ************

HG              GGGCAATCTTATGTTGAGGCTCAAGATAATTTAACTGTGCAAGAGTGGATGAGA
HUG             GGGCAATCTTATGTTGAGGCTCAAGATAATTTAACTGTGCAAGAGTGGATGAGA
XG              GGGCAATCTTATGTTGAGGCTCAAGATAATTTAACTGTGCAAGAGTGGATGAGA
TG              GGGCAATCCTATGTTGAGGCTCAAGATAATTTAACTGTGCAAGAGTGGATGAGA
                ******** *********************************************
```

(b)

```
tg              GGATATGGGCTATTTTAAGGAACAACGAGATGCTTACTTGGCCAGAGAAAATTAAATTTG
xg              GGATATGGGCTATTTTAAGGAACAACGAGATGCTTACTTGGCCTGAGAAAATTAAATTTG
hg              GGATATGGGCTATTTTAAGGAACAACGAAATGCTTACTTGGCCAGAGAAAATTAAATTTG
hug             GGATATGGGCTATTTTAAGGAATAACGAGATGCTTACTTGGCCAGAGAAAATTAAATTTG
                ********************** ***** ************** ****************

tg              CAATTGGGCTCCTGCCAGCAATGCTTGGTGGGCAATCCTATGTTGAGGCTCAAGATAATT
xg              CAATTGGGCTCCTGCCAGCAATGCTTGGTGGGCAATCTTATGTTGAGGCTCAAGATAATT
hg              CAATTGGGCTCCTGCCGGCAATGCTTGGTGGGCAATCTTATGTTGAGGCTCAAGATAATT
hug             CAATTGGGCTCCTGCCGGCAATGCTTGGTGGGCAATCTTATGTTGAGGCTCAAGATAATT
                **************** ******************** **********************

tg              TAACTGTGCAAGAGTGGATGAGAAGTCGGG
xg              TAACTGTGCAAGAGTGGATGAGAAGTCGGG
hg              TAACTGTGCAAGAGTGGATGAGAAGTCGGG
hug             TAACTGTGCAAGAGTGGATGAGAAGTCGGG
                ******************************
```

(c)

```
HG      ATGCTTACTTGGCCAGAGAAAATTAAATTTGCAATTGGGCTCCTGCCGGCAATGCTTGGT
HUG     ATGCTTACTTGGCCAGAGAAAATTAAATTTGCAATTGGGCTCCTGCCGGCAATGCTTGGT
XG      ATGCTTACTTGGCCTGAGAAAATTAAATTTGCAATTGGGCTCCTGCCAGCAATGCTTGGT
TG      ATGCTTACTTGGCCAGAGAAAATTAAATTTGCAATTGGGCTCCTGCCAGCAATGCTTGGT
        ************** ******************************** ************

HG      GGGCAATCTTATGTTGAGGCTCAAGATAATTTAACTGTGCAAGAGTGGATGAGAAGTCGG
HUG     GGGCAATCTTATGTTGAGGCTCAAGATAATTTAACTGTGCAAGAGTGGATGAGAAGTCGG
XG      GGGCAATCTTATGTTGAGGCTCAAGATAATTTAACTGTGCAAGAGTGGATGAGAAGTCGG
TG      GGGCAATCCTATGTTGAGGCTCAAGATAATTTAACTGTGCAAGAGTGGATGAGAAGTCGG
        ******** ***************************************************

HG      GGAGTACCTGATCGTGTAACAACAGAGGTGTTTATTGCTATGTCAAAGGCTCTGAACTTC
HUG     GGAGTACCTGATCGTGTAACAACAGAGGTGTTTATTGCTATGTCAAAGGCTCTGAACTTC
XG      GGAGTACCTGATCGTGTAACAACAGAGGTGTTTATTGCTATGTCAAAGGCTCTGAACTTC
TG      GGGGTACCTGATCGTGTGACAACGGAGGTGTTTATTGCTATGTCAAAGGCTCTGAACTTC
        ** ************** ***** ************************************

HG      ATTAACCCCGATGAACTTTCTATGCAATGCATT
HUG     ATTAACCCCGATGAACTTTCTATGCAATGCATT
XG      ATTAACCCCGATGAACTTTCTATGCAATGCATT
TG      ATTAACCCCGATGAACTTTCTATGCAATGCATT
        *********************************
```

(d)

```
hg      TTTGGGGCTTATCCCAATGTGCAGAACTTGTTTGGAGAACTTGGAATCAATGACCGATTG
hug     TTTGGGGCTTATCCCAATGTGCAGAACTTGTTTGGAGAACTTGGAATCAATGACCGATTG
tg      TTTGGGGCTTATCCCAATGTGCAGAACTTGTTTGGAGAACTTGGAATCAATGACCGATTA
xg330   TTTGGGGCTTATCCCAATGTGCAGAACTTGTTTGGAGAACTTGGAATCAATGACCGATTG
        *********************************************************** 

hg      CAGTGGAAGGAACATTCAATGATATTTGCTATGCCAAACAAGCCGGGGGAGTTCAGCCGA
hug     CAGTGGAAGGAACATTCAATGATATTTGCTATGCCAAACAAGCCGGGGGAGTTCAGCCGA
tg      CAGTGGAAGGAACATTCAATGATATTTGCTATGCCAAACAAGCCGGGGGAGTTCAGCCGA
xg330   CAGTGGAAGGAACATTCAATGATATTTGCTATGCCAAACAAGCCGGGGGAGTTCAGTCGA
        ******************************************************** ***

hg      TTTGATTTCCCTGAAAAACTTCCTGCACCTATAAATG-----------------------
hug     TTTGATTTCCCTGAAAAACTTCCTGCACCTATAAATG-----------------------
tg      TTTGATTTCCCTGAAAAACTTCCTGCACCCATAAATG-----------------------
xg330   TTTGATTTCCCTGAAAAACTTCCTGCACCCCGTAAACGCTTTCCTAAACTTTCAGAGTTA
        *****************************  **** *

hg      -------GGATATGGGCTATTTTAAGGAACAACGAAATGCTTACTTGGCCAGAGAAAATT
hug     -------GGATATGGGCTATTTTAAGGAACAACGAAATGCTTACTTGGCCAGAGAAAATT
tg      -------GGATATGGGCTATTTTAAGGAACAACGAGATGCTTACTTGGCCAGAGAAAATT
xg330   CTGACAGGGATATGGGCTATTTTAAGGAACAACGAGATGCTTACTTGGCCTGAGAAAATT
               **************************** ************** *********

hg      AAATTTGCAATTGGGCTCCTGCCGGCAATGCTTGGTGGGCAATCTTATGTTGAGGCTCAA
hug     AAATTTGCAATTGGGCTCCTGCCGGCAATGCTTGGTGGGCAATCTTATGTTGAGGCTCAA
tg      AAATTTGCAATTGGGCTCCTGCCAGCAATGCTTGGTGGGCAATCCTATGTTGAGGCTCAA
xg330   AAATTTGCAATTGGGCTCCTGCCAGCAATGCTTGGTGGGCAATCTTATGTTGAGGCTCAA
        *********************** ******************** ***************

hg      GATAATTTAACTGTGCAAGAGTGGATGAGA
hug     GATAATTTAACTGTGCAAGAGTGGATGAGA
tg      GATAATTTAACTGTGCAAGAGTGGATGAGA
xg330   GATAATTTAACTGTGCAAGAGTGGATGAGA
        ******************************
```
