# Supplementary figures and images for: A cucumber green mottle mosaic virus vector for virus-induced gene silencing in cucurbit plants
Source: Plant Methods. 2020 Feb 3;16:9. doi: 10.1186/s13007-020-0560-3 (PMC6996188; doi:10.1186/s13007-020-0560-3)

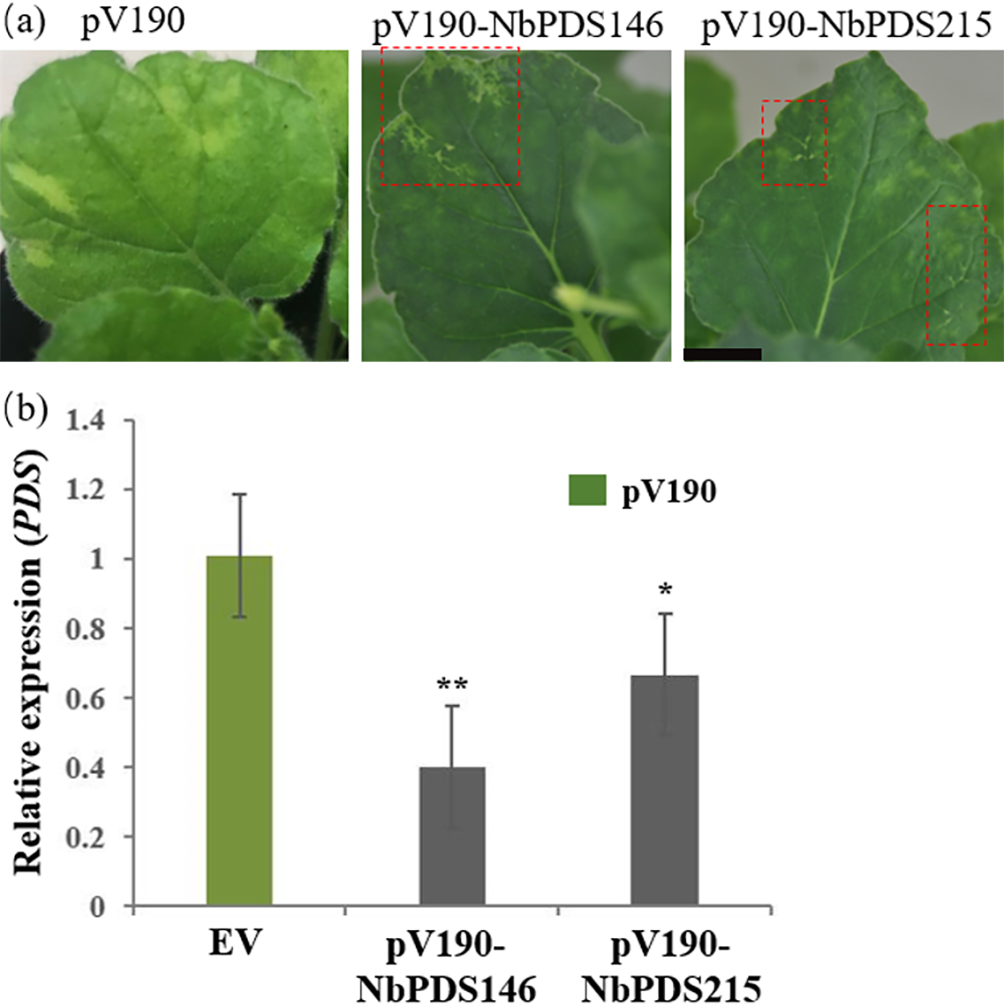

Supplement: Supplementary file 3 — Additional file 3: Fig. S2. Silencing efficiency of different length inserts (PDS) using the pV190 VIGS vector in N. benthamiana. Fragments of 146 bp, 215 bp were separately cloned into pV190 VIGS vector. (a) The silencing phenotypes were observed at 14dpi. Bar = 1 cm. (b) The relative expression level of PDS mRNA determined by real-time qRT-PCR. [file 13007_2020_560_MOESM3_ESM.png]
