# Supplementary material for: A cucumber green mottle mosaic virus vector for virus-induced gene silencing in cucurbit plants
Source: Plant Methods. 2020 Feb 3;16:9. doi: 10.1186/s13007-020-0560-3 (PMC6996188; doi:10.1186/s13007-020-0560-3)
Supplement: Supplementary file 4 — Additional file 4: Table S1. Primers used in this study. [file 13007_2020_560_MOESM4_ESM.doc]

**Supporting Information**

**Table S1.** Primers used in this study.

| **Primer name** | **sequence (5’-3’)** |
| --- | --- |
| **DelHindIII-X** | **GCATGCCTGCAGTCAACATGGTGG** |
| **DelHindIII-S** | **TGACTGCAGGCATGCGCTTAAAACC** |
| **S159Z-X** | **AGAAAGCTTAGTTTCGAGGGTCTTCT** |
| **S159Z-S** | **CGAAACTAAGCTTTCTAGGTGGTAGC** |
| **CP-TC-F** | **CTGTTTCTTTTGAAGACGGCTTACAAT** |
| **CP-TC-R** | **CGTCTTCAAAAGAAACAGAACTGGACTC** |
| **PXT1-F** | **ATGCCTGCAGTCAACATGGTGGAG** |
| **PXT1-R** | **CATGTTGACTGCAGGCATGCAAGC** |
| **27B-34-F** | **GGATCCGCTTCTGAAGAGTCCAGTT** |
| **27B-34-R** | **TTCAGAAGCGGATCCGCTAGGTGTG** |
| **58B-34-F** | **GGATCCGCTTCTGAAGAGTCCAGTTCTG** |
| **58B-34-R** | **CTCTTCAGAAGCGGATCCGAACATAAGAA** |
| **78B-34-F** | **ACTTAATGGATCCGCTTCTGAAGAGTCCAG** |
| **78B-34-R** | **GGATCCATTAAGTAAAGTCCTGACGGGAA** |
| **78B-99-F** | **ACTTAATGGATCCAGTTATAGGTCTAGGTCGCAG** |
| **78B-99-R** | **CTATAACTGGATCCATTAAGTAAAGTCCTGACGGGA** |
| **78-69P-X** | **GAGTGGATGAGACTCTTGCACAGTTAAATTATCTTGAGCCTCCAGTTATAGGTCTAGGTC** |
| **78-69P-S** | **GAGTCTCATCCACTCTTGCACAGTTAAATTATCTTGAGCCTCCATTAAGTAAAGTCCTG** |
| **CG-4F** | **GGATGTGGTCTATGGGTC** |
| **CG-4R** | **TGGAGATGCCATGCCGACCCTGGGCCCCTACCCGGG** |
| **3R** | **GACCCATAGACCACATCC** |
| **TxR~R** | **GGGTCGGCATGGCATCTCCAC** |

**Table S1 Continued**

| **Primer name** | **sequence (5’-3’)** |
| --- | --- |
| **CuPDS-HindIII-F** | **TCAGAGGCTACCACCTAGAAAGCTTATGCTTACTTG** |
| **CuPDS-HindIII-R** | **TCAGAAGACCCTCGAAACTAAGCTTTCTCATCCAC** |
| **CuPDS-HindIII-2F** | **TCAGAGGCTACCACCTAGAAAGCTTATGCTTACTTGGCCAGAG** |
| **CuPDS-HindIII-2R** | **TCAGAAGACCCTCGAAACTAAGCTTAATGCATTGCATAGAAAGTTC** |
| **CuPDS-HindIII-3F** | **TCAGAGGCTACCACCTAGAAAGCTTTGGGGCTTATCCCAA** |
| **CuPDS-HindIII-3R** | **TCAGAAGACCCTCGAAACTAAGCTTCTCATCCACTCTTGC** |
| **58-150-F** | **AGTGCTTCTTATGTTCGGATCCGGATATGGGCTATTTTAAGGA** |
| **58-150-R** | **GGACTCTTCAGAAGCGGATCCCCCGACTTCTCATCCACT** |
| **78-34-150F** | **GGACTTTACTTAATGGATCCGGATATGGGCTATTTTAAGGA** |
| **78-34-150R** | **CTGGACTCTTCAGAAGCGGATCCCCCGACTTCTCATCCACT** |
| **78-150-F** | **CCCGTCAGGACTTTACTTAATGGATCCGGATATGGGCTATTTTAAGGA** |
| **78-150-R** | **CGACCTAGACCTATAACTGGATCCCCCGACTTCTCATCCACT** |
| **58-213-F** | **AGTGCTTCTTATGTTCGGATCCATGCTTACTTGGCCAGAG** |
| **58-213-R** | **GGACTCTTCAGAAGCGGATCCAATGCATTGCATAGAAAGTTC** |
| **78-34-213F** | **GGACTTTACTTAATGGATCCATGCTTACTTGGCCAGAG** |
| **78-34-213R** | **CTGGACTCTTCAGAAGCGGATCCAATGCATTGCATAGAAAG** |
| **78-213-F** | **CCCGTCAGGACTTTACTTAATGGATCCATGCTTACTTGGCCAGAG** |
| **78-213-R** | **CGACCTAGACCTATAACTGGATCCAATGCATTGCATAGAAAGTTC** |
| **78-300-F** | **CCCGTCAGGACTTTACTTAATGGATCCTTTGGGGCTTATCCCAA** |
| **78-300-R** | **CGACCTAGACCTATAACTGGATCCTCTCATCCACTCTTGC** |
| **78-146N-F** | **CCCGTCAGGACTTTACTTAATGGATCCCTATTGGACTCTTGCC** |
| **78-146N-R** | **CGACCTAGACCTATAACTGGATCCGTTAAGTGCCTTTGAC** |
| **78-215N-F** | **CCCGTCAGGACTTTACTTAATGGATCCATGCAGAACCTGTTTGG** |
| **78-215N-R** | **CGACCTAGACCTATAACTGGATCCGGCAAGAGTCCAATAG** |
| **CuPDS-679F** | **TGTGTGGATTACCCTAGACC** |
| **CuPDS-906R** | **CCAAGCTGCTACCTTTCCAC** |

**Table S1 Continued**

| **Primer name** | **sequence (5’-3’)** |
| --- | --- |
| **wate-q-F** | **TGCTTTAGCGTTTTGGGGGA** |
| **wate-q-R** | **GACGTGCAGAAGCACGAAAA** |
| **cumsactin-F** | **ATGGTCAAGGCTGGATTTGC** |
| **cumsactin-R** | **TGAGCTTCATCACCAACATAGGC** |
| **NbPDS-qF** | **TGTGAACCCTGTCGGCCCTTG** |
| **NbPDS-qR** | **TTCTGGCTCCGGCCAAGAAGT** |
| **5574F** | **TCCTTTGACTTTAGAGGTCG** |
| **3UTR** | **TGGGCCCCTACCCGGGGAA** |
